# Comprehensive analysis of immune cell infiltration and role of MSR1 expression in aneurysmal subarachnoid haemorrhage

**DOI:** 10.1111/cpr.13379

**Published:** 2022-12-14

**Authors:** Xing Wang, Dingke Wen, Chao You, Chuanyuan Tao, Lu Ma

**Affiliations:** ^1^ Department of Neurosurgery, West China hospital Sichuan University Chengdu Sichuan China; ^2^ West China Brain Research Centre Sichuan University Chengdu Sichuan China

## Abstract

Aneurysmal subarachnoid haemorrhage (aSAH), resulting from the rupture of intracranial aneurysms, can yield high mortality and disability. This study aimed to explore the immune infiltration of aneurysmal tissues and investigate a novel mechanism underlying aSAH. We downloaded datasets containing expression profiles of aneurysmal and normal arterial tissues from the online database. Then a comprehensive bioinformatic strategy was conducted to select the biomarkers of aneurysmal tissues. Two calculation algorithms were performed to identify the unique immune characteristics between aneurysmal tissues and normal arteries. Double immunofluorescence staining was used to investigate the role of pathway‐related proteins in the inflammatory process after aSAH. Six microarray datasets were integrated, and another RNA‐sequencing dataset was used as the validation dataset. Functional enrichment analysis of the differentially expressed genes indicated that immune‐related processes were closely related to the progression of aSAH. We then performed immune microenvironment infiltration analysis, and the results suggested macrophages were abnormally enriched in aneurysmal tissues. Core gene MSR1 was filtered through a comprehensive bioinformatic strategy. Our analysis suggested that MSR1 might be associated with macrophage activation and migration. Our study elucidated the impact of macrophage and MSR1 on aSAH progression. These findings were helpful in gaining insight into the immune heterogeneity of aneurysmal tissues and normal arteries, and in identifying patients who might benefit from immunotherapy.

## Graphical abstract

In this study, we performed extensive bioinformatic analysis to investigate the immune infiltration and identify related biomarkers in patients with aneurysmal subarachnoid haemorrhage (aSAH). Our study elucidated the impact of macrophage on aSAH progression. Besides, MSR1 was identified as a biomarker involved in the inflammatory reactions. This study may be of benefit to patients who are at high risk of suffering aSAH which allows for timely diagnosis and potential therapy target.

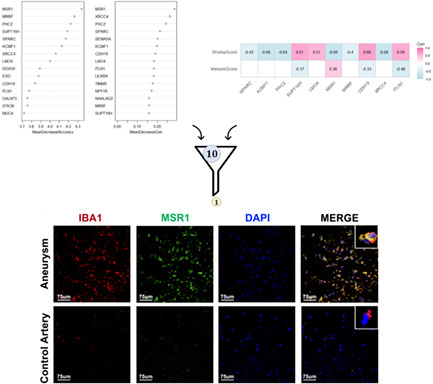

## 1 INTRODUCTION

Aneurysmal subarachnoid haemorrhage (aSAH) is a deadly cerebrovascular disease.^1^, ^2^, ^3^ It is estimated that more than 25% of patients with aSAH died in the hospital, and more than 40% have persistent neurological deficits.^2^ Moreover, patients who survive the initial haemorrhage are susceptible to cerebrovascular diseases and have mortality rates that exceed the general population.^3^, ^4^ For example, the recurrence of aSAH in survivors was estimated to be 15‐times higher than that in the general population. Thus, it is critical to recognize the cellular and molecular differences between aneurysmal tissues from aSAH patients and normal intracranial arterial tissues, which could offer a theoretical basis for the high recurrence of cerebrovascular disease in patients with aSAH.

Inflammatory reactions are involved in the formation, progression and deterioration of aSAH.^5^ Once the intracranial aneurysm (IA) ruptures, immune cells, haemoglobin and cytokines enter the central nervous system and mediate inflammatory responses.^6^, ^7^ The abnormal immune responses after aSAH have been proposed to be a critical component in the development of secondary brain injury.^8^, ^9^, ^10^ At this point, researchers are inclined to believe that aSAH is rather a chronic disease that is caused by a massive systemic inflammatory response than a one‐off event.

Among these immune reactions, evidence indicates that macrophages play a particular role in SAH‐induced inflammation.^11^, ^12^ However, previous studies in the same field were conducted with small sample sizes, and their findings need to be confirmed in large cohorts and comprehensive bioinformatics analyses. To identify the potential biomarkers related to the pathogenesis of aSAH that might offer novel molecular features, we performed this study to clarify the cellular and molecular processes implicated in the development of aSAH, which might facilitate the timely diagnosis and subsequent treatment for this fatal disease. Our findings may also provide a theoretical basis for the high incidence of intracranial vascular disease after ASAH.

## 2 MATERIALS AND METHODS

### 2.1 Data acquisition and integration

We systematically searched Gene Expression Omnibus (GEO) database with the following keywords, ‘subarachnoid haemorrhage’, ‘intracranial aneurysm’, ‘cerebral aneurysm’ and so forth. Ultimately, seven available transcriptome data of tissue samples from aneurysmal walls and normal intracranial arteries were obtained; six were microarray datasets (GSE6551, GSE13353, GSE15629, GSE26969, GSE54083 and GSE75436). The detailed information of each dataset was presented in Table S1. Most studies collected ruptured IA tissues within 24 hours after initial bleeding. The integrated dataset contained 27 IA tissues from patients with aSAH and 38 normal arteries. The R package ‘sva’ was applied to remove the batch effect. We also obtained one RNA‐sequencing (RNA‐seq) dataset (GSE122897) as the validation dataset, which contained 23 aneurysmal tissues from patients with aSAH and 16 normal arteries tissues. Additionally, five pairs of aneurysmal tissues from aSAH patients and superficial temporal arteries from patients who were free of vascular diseases during surgery were obtained from our hospital. This study was based on the ethical principles of the Helsinki Declaration and approved by the ethical committee of the West China Hospital of Sichuan University.

### 2.2 Immunofluorescence assay

The detailed procedures have been described in the previous study.^13^ Briefly, tissues were sliced into 5‐μm‐thick frozen sections. Next, after blocking for 1 hour with 5% bovine serum albumin, primary antibodies, including rabbit anti‐MSR1 (ABclonal, A2401), mouse anti‐IBA1 (Abcam, ab283319), rabbit anti‐CD86 (ABclonal, A19026) were incubated with the samples overnight at 4°C. Then, the primary antibodies were washed three times by TBS. Secondary antibodies, including the goat anti‐rabbit antibody (AS011, ABclonal) and the goat anti‐mouse antibody (AS001, ABclonal) were placed on the slides and incubated for 1 hour at 25°C. The antibody dilution ratio was following the online instructions. The sections were subsequently stained with DAPI for nuclear labelling. Images were analysed by Image J 1.4 software.

### 2.3 Enrichment analysis

Differentially expressed genes (DEGs) between two clusters were defined as those genes with adjusted *p* values <0.05 and absolute values of log2(foldchange) >0.50. DEGs were obtained from microarray datasets and RNA‐seq dataset using the ‘limma’ and ‘DESeq2’ packages, respectively. The Gene Ontology (GO) pathway enrichment of DEGs was conducted with the DAVID database (https://david.ncifcrf.gov/).^14^ A false discovery rate (FDR) value <0.05 was deemed statistically relevant. The procedures were achieved by ‘clusterProfiler’ package, and visualization was completed by the ‘ggplot2’ package.

### 2.4 Gene set variation analysis

We downloaded the signature ‘c7.all.v7.4.symbols_immune’ from the Molecular Signatures Database (MSigDB).^15^ Differential pathways between different groups were screened by the ‘limma’ package. The results were presented in the heatmap. Pathways with adjusted *p* values smaller than 0.05 were deemed as statistically relevant. The procedures were achieved using the R package ‘GSVA’.

### 2.5 Immune infiltration analysis

The infiltration profiles of different immune cells were assessed by the Microenvironment Cell Populations‐counter (MCPcounter) algorithm and the Xcell algorithm. The MCPcounter algorithm is an approach for measuring the comparative enrichment of 10 types of immune cells, including bone marrow dendritic cells, neutrophils, NK cells, B lineage, fibroblasts, T cells, monocyte lineage, CD8 T cells, cytotoxic T cells and endothelial cells in various tissue samples using labelled genes that were optimized for interrogation of transcriptomic data.^16^ In addition, Xcell algorithm was applied to determine the proportion of infiltrating immune cell subsets in different tissues. This tool could provide independent enrichment scores across 64 immune and stroma cell types based on intrinsic algorithms.^17^

### 2.6 Additional analyses

The random forest approach was used to identify the most critical candidate genes associated with distinct characteristics. In brief, a set of concerned genes were employed as the input for the construction of prediction models. Preferred genes were chosen depending on the mean decrease Gini and mean decrease accuracy. To compare the predictive sensitivity of the selected genes, area under the curve (AUC) values obtained from the receiver operating characteristic (ROC) curves were calculated. The above analyses were done with the ‘randomForest’ and ‘pROC’ packages.

The Wilcoxon test compared the expression level of genes between aneurysmal tissues and normal intracranial arteries. We applied the Pearson correlation test to evaluate the linear relationship between different genes. The Spearman correlation analysis was constructed to determine the relationships between selected genes and various immune cells. The statistical analyses were carried out with the R software (version 4.0.5). All tests were conducted to be two‐sided, and a *p*‐value smaller than 0.05 revealed a statistically significant difference.

## 3 RESULTS

### 3.1 Functional enrichment analysis of DEGs

**FIGURE 1:**
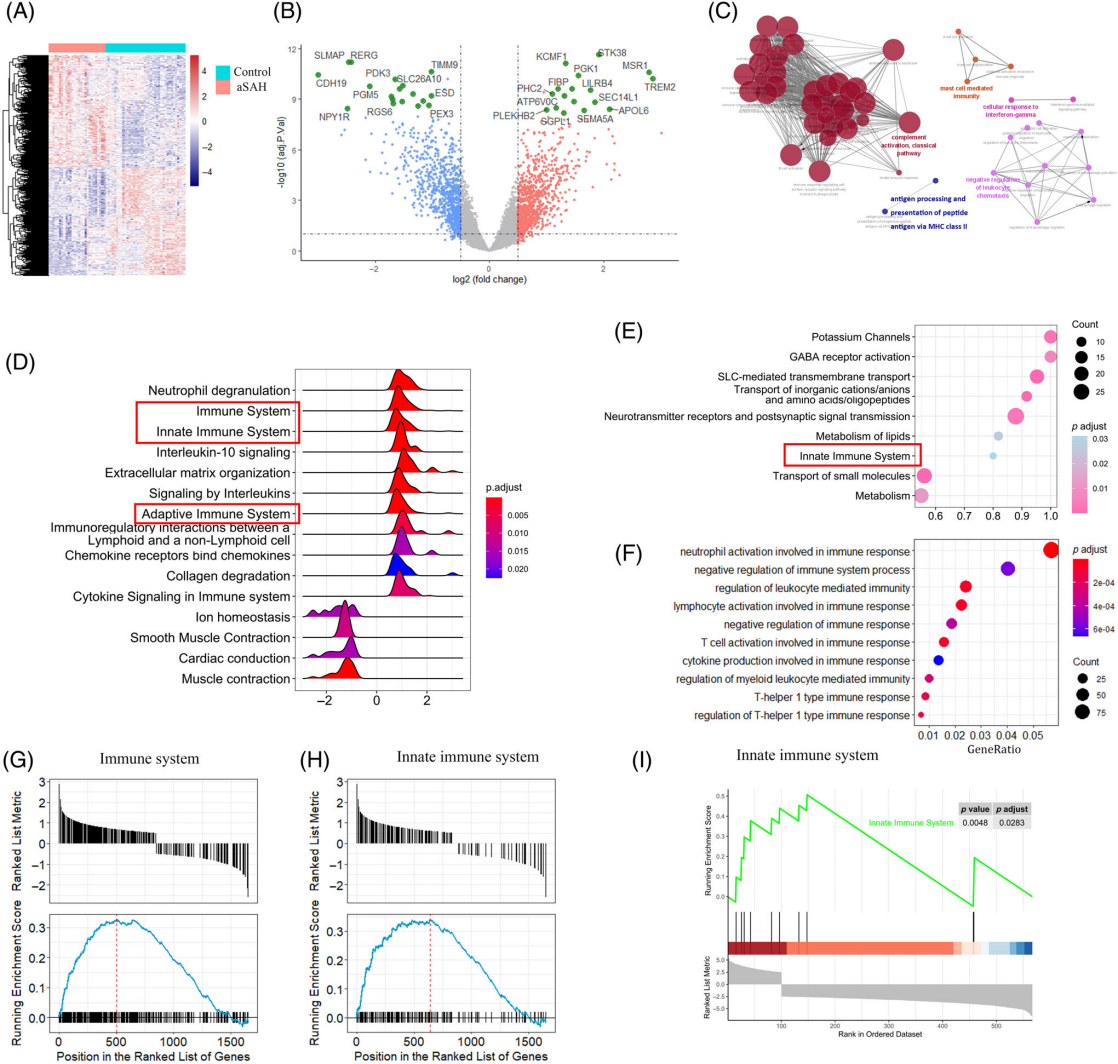
Acquisition of DEGs and enrichment analysis in the integrated and validation cohorts. (A) Clustering plot presenting the expression landscape between aneurysmal tissues and normal arteries. (B) Volcano plot of DEGs in the two groups from the integrated dataset. (C) Network interactions analysed by Cytoscape software using the validation dataset. (D) The top 15 reactome enrichment terms of aneurysmal tissues and normal arteries. (E) GSEA of the validation dataset showing innate immune system was significantly enriched. (F) GO functional enrichment terms involved in the immune‐related process. (G and H) Representative enriched pathways of GSEA in the integrated dataset. The results of immune system and innate immune system were presented. (I) GSEA of the validation dataset showing innate immune system involved in the enriched pathways. DEGs, differentially expressed genes; GESA, gene set enrichment analyses; GO, gene ontology

The cutoff criteria determined according to an adjusted *p* value smaller than 0.05 and an absolute value of log2(foldchange) larger than 0.5. Overall, after data merging and removal of batch effects, a total of 1649 genes were regarded as DEGs, among which 846 were upregulated and 803 genes were downregulated. We performed hierarchical clustering analysis to visualize the distinguishment ability of DEGs between aneurysmal tissues and control arteries (Figure 1A). Volcano plot was used to exhibit a group of genes that were significantly highly and lowly expressed (Figure 1B). The mutual upregulated and downregulated DEGs were then uploaded to the R software in order to further inform the potential functions of genes in the progression and rupture of IA. DEGs from the validation dataset were also obtained between aneurysmal tissues and normal arterial tissues. To enable visualization of the network interactions among the potential biological processes, the set of genes were imported into the Cytoscape software. The results suggest that activation of inflammatory signalling pathways is engaged in the progression of aSAH.

To further explore the enrichment pathway of DEGs, the top 15 enrichment terms according to the enrichPathway function were presented as ridge plot. According to the analysis, immunological processes including innate and adaptive immune systems were significantly upregulated. The results were showed in Figure 1D. Similar results were obtained from the validation dataset (Figure 1E). The top 10 GO terms associated with immune response were therefore extracted in Figure 1F. Results of GSEA analysis suggested that the immune system and innate immune system were upregulated in aneurysmal tissues compared with normal arteries tissues (Figure 1G–I).

### 3.2 Immune profiles of aneurysmal and arteries tissues

**FIGURE 2:**
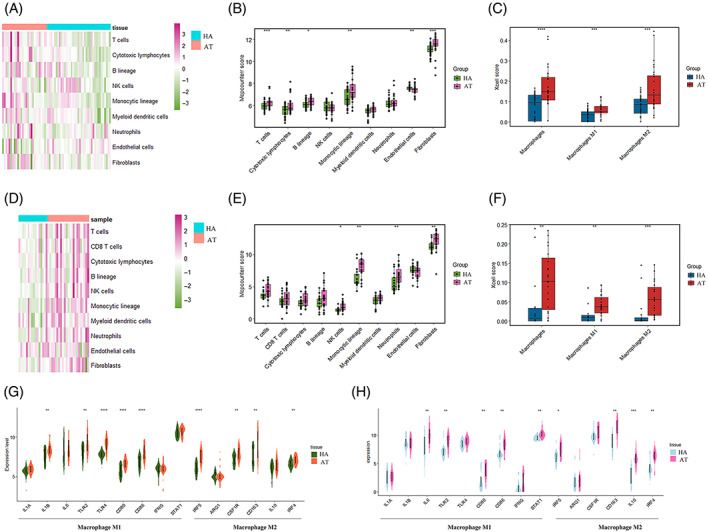
Immune profiles of aneurysmal tissues and normal arteries analysed by Xcell and Microenvironment Cell Populations‐counter (MCPcounter) approach. (A and B) The heatmap and boxplot illustrating the global enrichment of nine types of immune cells in the combined dataset using the MCPcounter algorithm. (C) Boxplot showing the Xcell enrichment score in macrophages, macrophages M1 and macrophages M2 in the integrated dataset. (D and E) The heatmap and boxplot illustrating the global enrichment profiles of immune cells in the validation dataset using the Xcell algorithm. (F) The boxplot showing the Xcell enrichment score in macrophages, macrophages M1, and macrophages M2 in the validation dataset. (G and H) Expression profiles of the marked genes of macrophages M1 and M2 in the integrated dataset and validation dataset. Statistical significance was tested by the Wilcoxon test (****p < 0.0001; ***p < 0.001; **p < 0.01; *p < 0.05). AT, aneurysmal tissues; HA, healthy arteries

Given that the above analysis revealed that immune‐associated features and signalling pathways were mainly abundant in aneurysmal tissues, we assessed the profile of different immune cells in each tissue sample using MCPcounter and Xcell tools. The abundance profiles of nine types of immune cells were clustered as heatmap, as displayed in Figure 2A,B. The results showed monocytic lineage (macrophages and monocytes) were dominant in aneurysmal tissues. We further performed the Xcell algorithm to compare the enrichment scores of different types of macrophages. The results revealed that both macrophages M1 and M2 were enriched in aneurysmal tissues (Figure 2C). The comparison between aneurysmal tissues and normal arteries in the validation dataset is shown in Figure 2D,E. The findings were validated by the RNA‐seq dataset (Figure 2F). Several cell markers of macrophages M1 and M2 were then compared. These results showed that the expression levels of these genes in the aneurysmal tissues were substantially elevated in the aneurysmal tissues compared to the normal arteries (Figure 2G,H).

### 3.3 Identification of key genes based on random forest model and Xcell immunescore

**FIGURE 3:**
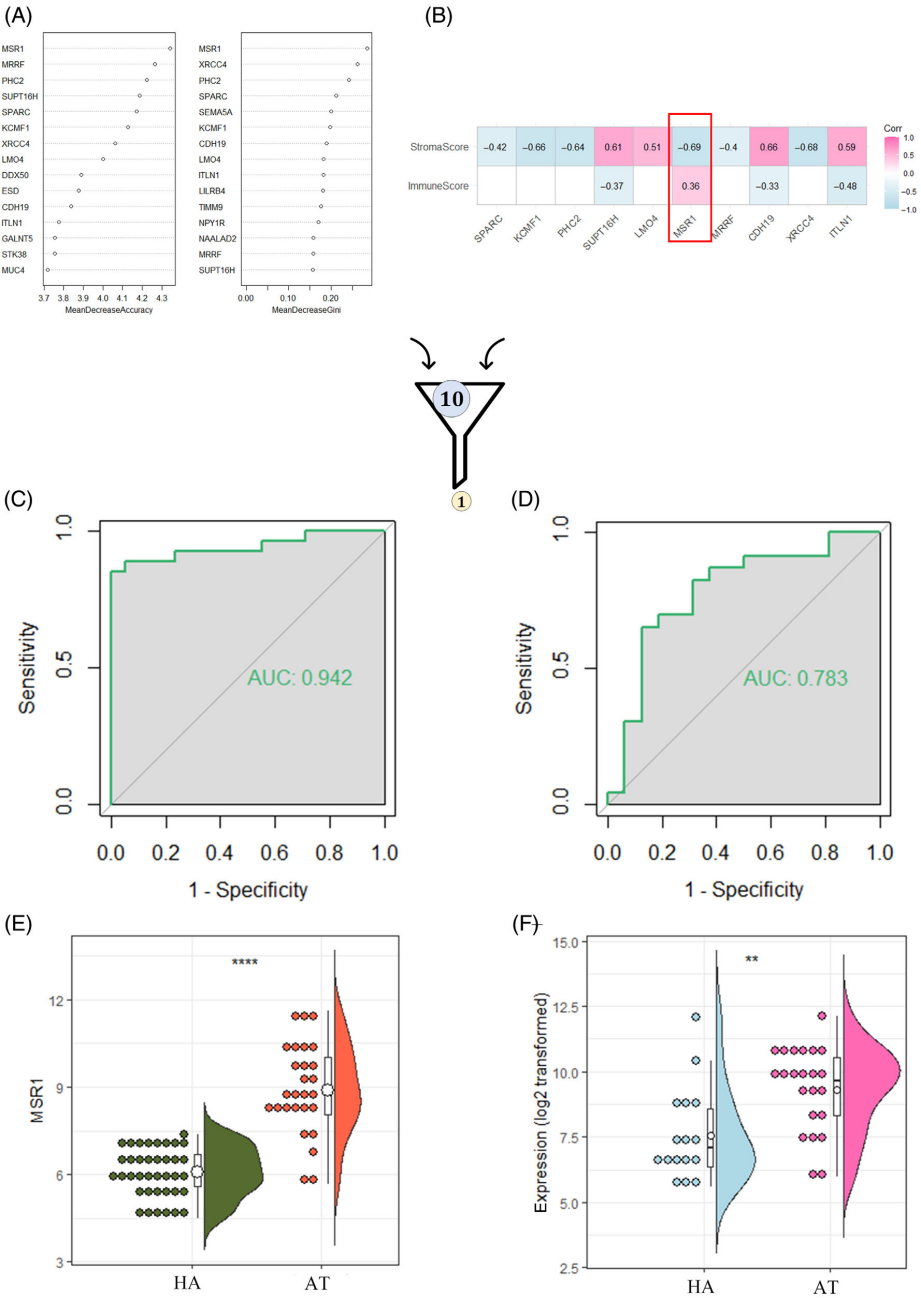
(A) Ten genes were overlapped in two ranking methods. (B) The heatmap showing the correlations between each concerned gene and immunescore. (C and D) The ROC analysis of MSR1 in the integrated dataset and validation dataset. The AUC was 0.942 and 0.783, respectively. (E and F) Expression profiles of MSR1 in the integrated dataset and validation dataset. AT, aneurysmal tissues; AUC, area under the curve; HA, healthy arteries; ROC, receiver operating characteristic

Random forest was conducted to filter the genes related to the above immune infiltration profiles. Two ranking methods were used to obtain the intersection (Figure 3A). For the concerned genes, we compared their correlation coefficient with stromascore and immunescore derived from Xcell algorithm. As shown in Figure 3B, MSR1 was significantly and positively correlated with immune score but negatively correlated with stroma score. To verify the predictive power of the selected gene, ROC curves were developed. Then AUC values were obtained to assess the predictive consistency and accuracy of the selected gene (Figure 3C,D). Eventually, MSR1 was chosen as a candidate gene for further investigation. The 95% confidence interval for AUC was 0.876–1.000, and the predicted AUC was 0.942, revealing the stability of MSR1 in discriminating aneurysmal tissues from normal arteries. The expression profile of MSR1 in the integrated and validation datasets was shown in Figure 3E,F. MSR1 was highly expressed in aneurysmal tissues.

### 3.4 Association of MSR1 and immune cell infiltration

**FIGURE 4:**
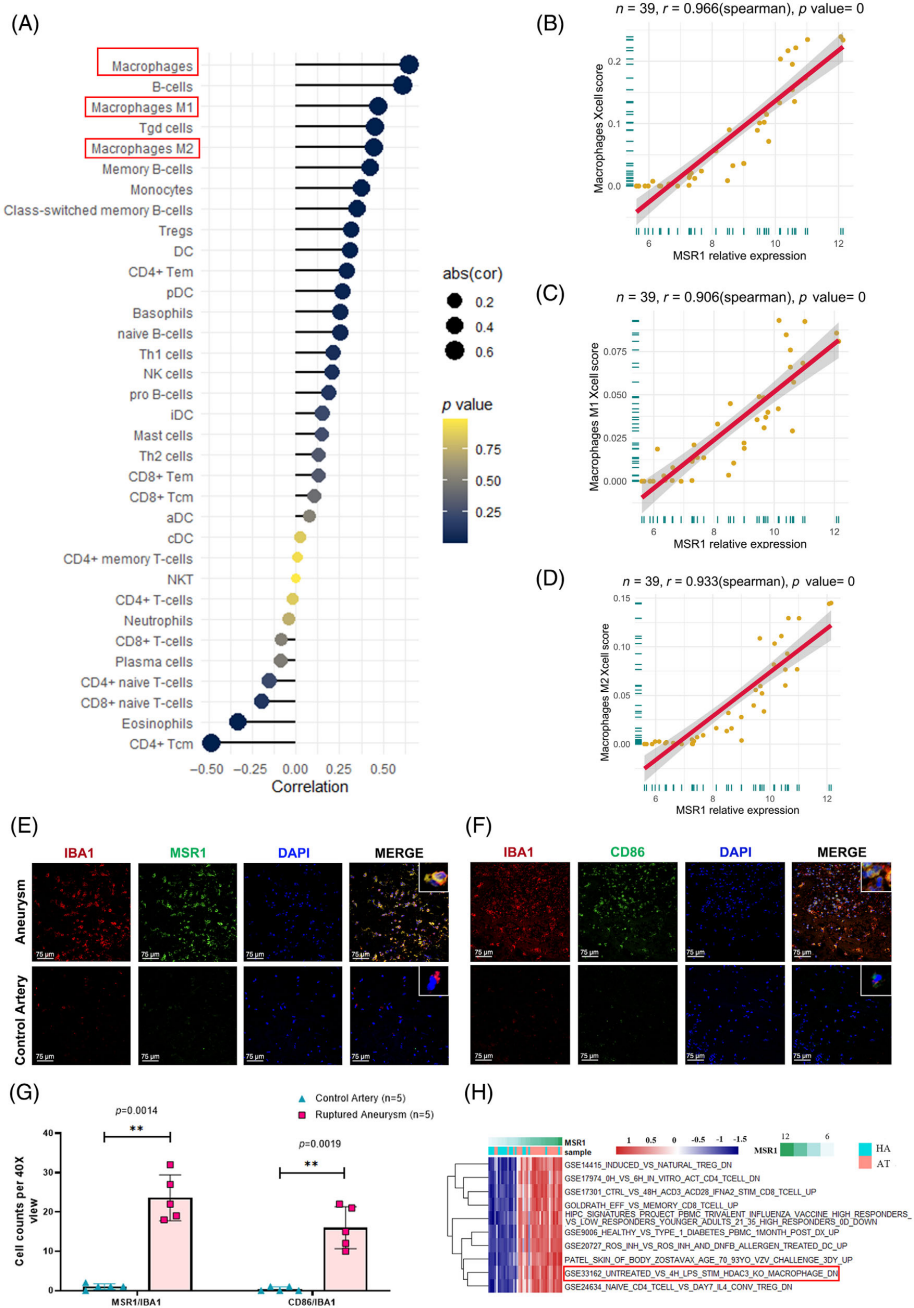
Spearman's correlations between enrichment scores of each immune cell types in Xcell approach and MSR1 expression levels in the (A) integrated dataset and (B–D) validation dataset. The correlation coefficient between MSR1 and macrophages, macrophages M1, macrophages M2 was 0.966 (*p* < 0.001), 0.906 (*p* < 0.001) and 0.933 (*p* < 0.001) in the validation dataset, respectively. (E and F) Immunofluorescence staining showing MSR1 (green), IBA1 (red), CD86 (green) and DAPI (blue) in aneurysmal tissues and normal arteries. Scale bar = 75 μm. (G) Quantitative analysis of IBA1, MSR1, CD86 and DAPI expression in arterial samples in aneurysmal group (*n* = 5) and normal arteries group (*n* = 5). Aneurysm represents ruptured aneurysmal tissue and control artery represents normal middle meningeal artery. (H) Significant immune‐related pathways analysed by GSVA between aneurysmal tissues and normal arteries along with MSR1 expression. GSVA, gene set variation analysis

Infiltration of 34 types of immune cells in the integrated dataset was analysed using the Xcell tool. The association between MSR1 and the infiltration landscape of immune cells was evaluated by the Spearman correlation test. Among all the immune cells, MSR1 expression was strongly correlated with macrophages (correlation coefficient: 0.652; *p* < 0.001; Figure 4A). The expression level of MSR1 was also significantly associated with macrophages group 1 (correlation factor: 0.474; *p* < 0.001) and group 2 (correlation factor: 0.449; *p* < 0.001). Similar results were observed in the RNA‐seq dataset (Figure 4B–D). The expression profiles of MSR1 and cell markers of macrophages were investigated by immunofluorescence double‐staining. The results revealed that almost all MSR1‐positive cells were also stained for IBA1 and CD86 (Figure 4E–G). Gene set variation analysis indicated macrophage‐related pathways activated along with the upregulation of MSR1 expression (Figure 4H).

### 3.5 Biofunction prediction of MSR1

**FIGURE 5:**
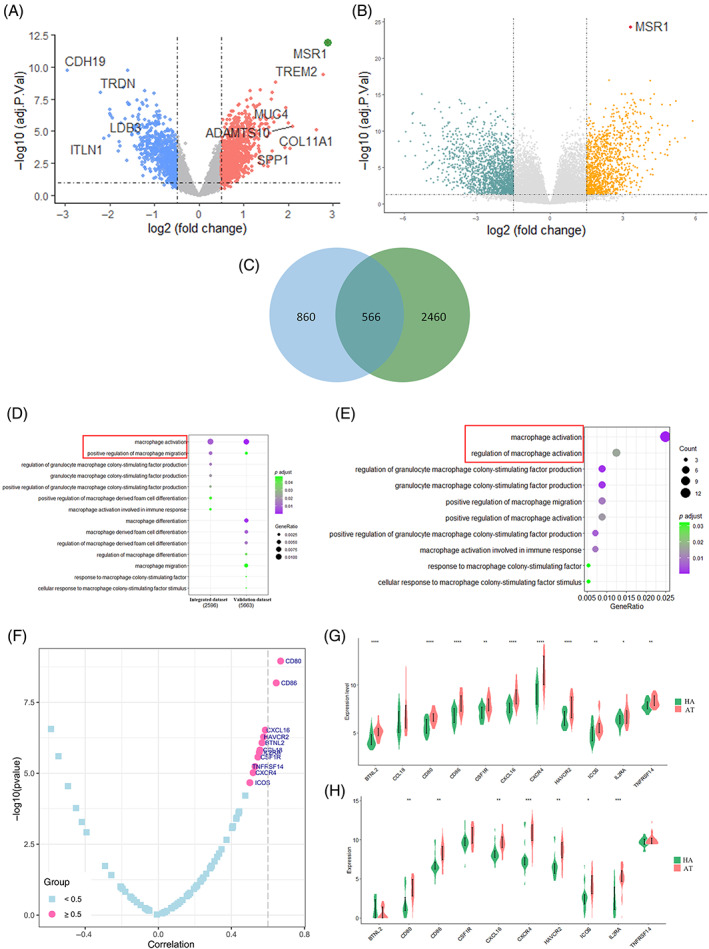
DEGs identified in the (A) integrated dataset and (B) validation dataset according to MSR1 expression. (C) A total of 566 genes are obtained by intersecting DEGs from different datasets. (D) GO analysis of the intersecting DEGs. (E) GO analysis of DEGs obtained from different datasets respectively in immune‐related enrichment terms. (F) Associations among MSR1 with a set of immunomodulators. (G and H) The expression profiles of a set of immunomodulators with correlation coefficients larger than 0.5 between aneurysmal tissues and healthy arteries in the integrated dataset and validation dataset. Statistical significance was tested by the Wilcoxon test (****p < 0.0001; ***p < 0.001; **p < 0.01; *p < 0.05). AT, aneurysmal tissues; DEGs, differentially expressed genes; GO, gene ontology; HA, healthy arteries

To further discover the potential mechanisms of macrophage regulation by MSR1, DEGs between MSR1 low and high‐expression groups were obtained in the combined dataset and the validation dataset (Figure 5A,B). The intersection of DEGs was then obtained for further functional enrichment analysis (Figure 5C). Considering the above results, GO analysis was performed to predict the biological functions of MSR1 related to macrophages (Figure 5D). We also performed GO analysis using the intersection of DEGs (Figure 5E). The results demonstrated that higher MSR1 expression was associated with macrophage activation and migration.

Additionally, various immunomodulators were downloaded from the TISIDB database. Pearson correlation test was then conducted to investigate the correlations between MSR1 and each immunomodulator. Immunomodulatory factors with correlation coefficients larger than 0.5 with MSR1 were CD80, CD86, CXCL16, HAVCR2, BTNL2, CCL18, IL2RA, CSF1R, TNFRSF14, CXCR4 and ICOS (Figure 5F). These factors were highly expressed in aneurysmal tissues, and the results were validated by the RNA‐seq dataset (Figure 5G,H).

## 4 DISCUSSION

This study indicated that macrophage‐mediated inflammation played a critical role in the progression of IAs. Our findings thus provided a theoretical basis for developing drug therapies to inhibit macrophage‐mediated inflammatory responses. Furthermore, MSR1 was identified through extensive bioinformatic analysis and considered to be closely involved in the immune microenvironment. Since MSR1 might impact the progression of aSAH by regulating macrophages, novel nanomaterials could be expected to intervene in this process.^18^, ^19^, ^20^ However, although we have discovered and validated the predictive value and potential biological function of MSR1 in aneurysmal tissues, future in vivo and in vitro research is still warranted to explore the upstream and downstream signalling pathways of MSR1.

It appears that IA formation, progression and rupture cannot be completely interpreted by a single mechanism.^21^ However, it is widely acknowledged that inflammation has a role in the formation and development of IA. Several studies have demonstrated that inflammatory cells, particularly macrophages, infiltrated walls of IAs in humans.^22^, ^23^ It seems that elevated levels of inflammation in aneurysms are related to the destruction and rupture of the aneurysm wall. In addition, recent evidence shows excessive neuroinflammation following aSAH results in second brain injury, leading to functional impairment and unfavourable outcomes.^24^, ^25^ Moreover, the administration of pharmacological agents with anti‐inflammatory properties decreases the risk of developing SAH from ruptured IAs. Commonly used medications in clinical practice are statins and non‐steroidal anti‐inflammatory drugs.^26^ Also, recent studies have observed the favourable effects of tetrahedral DNA in suppressing neuroinflammation and in treating neurological diseases.^27^, ^28^, ^29^, ^30^, ^31^

It is therefore important to thoroughly investigate the involvement of the immune landscape in aneurysmal tissues. In the present analysis, we have shown the critical roles of macrophages in the progression of aSAH by analysing the immune infiltration landscape of aneurysmal and normal arterial tissues. We used several recently developed algorithms to assess the immune cell infiltration landscape in aneurysmal tissues. First, we applied two algorithms and revealed that inflammatory cells, particularly macrophages, invaded the aneurysmal wall. Second, we identified key genes contributing to the molecular mechanism of IA progression and rupture based on feature elimination and immune scores. Third, the biological functions of essential genes and downstream signalling pathways were investigated and predicted. All the findings were validated by an independent dataset. However, these two algorithms, although helpful, cannot provide the expression levels of the concerned genes in different cells. To obtain the complete elaboration of the role of MSR1 in the progression of IA, single cell sequencing is required.

Macrophages of monocytic lineage represent an essential element of the innate immune system in the nervous system. They play a crucial part in the initiation, regulation and termination of inflammation due to their involvement with antigen presentation and cytokine production.^32^ As the majority of cellular components of leukocyte infiltration are associated with the risk of rupture.^33^ Findings from previous animal and human research suggested that macrophage‐mediated cellular and molecular inflammation is implicated closely in the development and rupture of IAs. For example, early evidence revealed loss of internal elastic lamina and extracellular matrix remodelling were the histological characteristic of IA.^34^, ^35^ The degenerative alterations in the tunica media in IA walls decrease arterial stiffness and increase the probability of rupture. Inspection of proteases in human aneurysmal tissue samples revealed elevated activity of matrix metalloproteinases (MMPs) in the aneurysmal walls than in the control arteries.^35^ Recent studies suggest the progression and rupture of IA may be regulated by MMPs generated from macrophages, particularly MMP‐2, MMP‐9.^36^, ^37^ Moreover, inhibitors of MMPs may inhibit the progression of IA.^38^ Meanwhile, macrophages act as critical contributors to pro‐inflammatory signalling. They secrete several factors related to the progression and rupture of IAs, including interleukin‐6 (IL‐6), interleukin‐1 beta (IL‐1𝛽), tumour necrosis factor‐alpha (TNF‐α), and so forth.^34^ Some cytokines can activate other inflammatory cells such as neutrophils and lymphocytes.

M1 and M2 macrophages have been recognized in IA specimens from human subjects.^39^, ^40^ Polarization of macrophages has a vital role in neuroinflammation. Macrophages in different polarization states might play a critical position in the development, growth and rupture of IAs by regulating inflammation and vascular remodelling, and may even play a neuroprotective role at different stages after aSAH.

Several human and animal studies endorse the upregulation and involvement of MSR1 in developing aneurysms. MSR1 primarily mediates oxidized lipoprotein uptake and facilitates foam cell production in atherosclerotic plaques. Reeps et al. have shown that MSR1 was significantly expressed in the abdominal aortic aneurysm (AAA) walls, and its expression level was associated with AAA diameter.^41^ Currently, Kong et al. have found that MSR1 promotes phagocytosis of myelin debris and generation of foam macrophage following spinal cord injury, contributing to the pro‐inflammatory polarization of macrophages.^42^ Regarding the mechanism, the MSR1‐mediated NF‐κB signalling pathway promotes the emission of inflammatory mediators, which subsequently leads to neuronal apoptosis. On the other hand, the role of MSR1 in tumours has been elucidated, particularly in gliomas. It has been reported that high expression of MSR1 was an independent prognostic factor of glioma.^43^ This marker gene is intimately associated with the tumour microenvironment and may play a key role in the immune therapy of glioma patients.

In addition, several immunomodulators significantly correlated with the expression of MSR1 were observed in the present bioinformatics analyses. Genes significantly positively correlated with MSR1 expression, primarily involved in macrophage activation and apoptotic responses. For example, CD80 and CD86 are markers of macrophage polarization towards M1, which are involved in macrophage activation and pro‐inflammatory responses. CXCL16 is a scavenger receptor on the surface of macrophages. This gene has been recognized to be involved in the pathophysiological process of atherosclerosis.^44^ Overexpression of CXC16 could also enhance apoptosis.^45^ HAVCR2 is also involved in regulating the activation of macrophages.^46^

## 5 CONCLUSIONS

Until now, the underlying mechanisms of IA progression and rupture have not been elucidated in detail. Our analysis provided extended information on the immune landscape of aneurysm tissues after aSAH. We also identified MSR1 as one of the critical genes involved in inflammatory responses. Our study may help elucidate the impact of macrophage on IA progression and identify biomarkers and therapeutic targets. Further evaluation of the role of MSR1 in brain injury and complications after aSAH is necessary through the application of genetic deletion in animal models of SAH to provide informative treatment options for patients with aSAH.

## AUTHOR CONTRIBUTIONS

*Conceptualization*: Xing Wang, Lu Ma and Chuanyuan Tao. *Methodology*: Xing Wang and Dingke Wen. *Software*: Xing Wang and Dingke Wen. *Validation*: Xing Wang, Dingke Wen and Chao You. *Formal analysis*: Xing Wang. *Writing—original draft preparation*: Xing Wang. *Writing—review and editing*: Xing Wang and Dingke Wen. *Visualization*: Lu Ma and Chuanyuan Tao. *Supervision*: Chuanyuan Tao, Lu Ma and Chao You. All authors have read and agreed to the published version of the manuscript.

## FUNDING INFORMATION

This research was funded by the National Key Research & Development Program of China (grant numbers: 2018YFA0108604 and 2018YFA0108603); Clinical Incubation Program of West China Hospital (grant number: 2018HXFH008); and National Natural Science Foundation of China (grant number: 82201450). The funders had no role in the design or conduct of this research.

## CONFLICT OF INTEREST

The authors declare that they have no known competing financial interests or personal relationships that could have appeared to influence the work reported in this article.

## Supporting information


**Table S1**. Recruit criterion of each dataset

## Data Availability

All data generated or analysed during this study are included in this article. The data sets supporting the conclusions of this article are available from the GEO database (https://www.ncbi.nlm.nih.gov/gds).
