# Supplementary material for: Comprehensive analysis of immune cell infiltration and role of MSR1 expression in aneurysmal subarachnoid haemorrhage
Source: Cell Prolif. 2022 Dec 14;56(6):e13379. doi: 10.1111/cpr.13379 (PMC10280136; doi:10.1111/cpr.13379)
Supplement: Supplementary file 1 — Table S1. Recruit criterion of each dataset [file CPR-56-e13379-s001.docx]

Supplemental Table 1 Recruit criterion of each dataset

| Dataset | Recruit criterion |
| --- | --- |
| GSE6551 | Intracranial arteries were collected from ruptured or unruptured IAs and from the contralateral vessel of the same patients to represent intracranial arteries. |
| GSE13353 | Fundi of rupturedand unruptured sIAs were resected after microsurgical clipping of the neck. |
| GSE15629 | Full-thickness vessel wall samples from 8 RIA and 6 UIA domes were prospectively collected from patients undergoing microsurgical clipping. Additionally, 5 middle meningeal artery segments were obtained during standard neurosurgical procedures. |
| GSE26969 | Intracranial aneurysms were obtained from patients during microsurgical clipping. All samples were unruptured aneurysms confirmed by magnetic resonance image or digital subtraction angiography. A set of 3 normal arteries (superficial temporal artery) was obtained from patients who were free of vascular diseases during surgery. |
| GSE54083 | Aneurysmal domes from 8 RIAs and 5 UIAs were harvested after surgical clipping. All the IAs were saccular. As a control, 10 superficial temporal arteries were obtained during standard neurosurgical procedures such as STA–middle cerebral artery bypass procedures. |
| GSE75436 | Unruptured intracranial aneurysm and superficial temporal artery |
| GSE122897 | Aneurysm tissue samples were excised after complete obliteration  of the aneurysm with a clip in patients aged ≥18 years undergoing neurosurgical clipping of a ruptured, or unruptured saccular intracranial aneurysm. Control tissue sample of an intracranial cortical artery was obtained from the resected brain tissue of patients aged ≥16 years who underwent surgery because of intractable epilepsy. |
